# *TUSC1*, a Putative Tumor Suppressor Gene, Reduces Tumor Cell Growth *In Vitro* and Tumor Growth *In Vivo*

**DOI:** 10.1371/journal.pone.0066114

**Published:** 2013-06-11

**Authors:** Zhihong Shan, Abbas Shakoori, Sohrab Bodaghi, Paul Goldsmith, Jen Jin, Jonathan S. Wiest

**Affiliations:** 1 Laboratory of Cancer Biology and Genetics, Center for Cancer Research, National Cancer Institute, Bethesda, Maryland, United States of America; 2 Advanced Genome Technology Center, Mayo Clinic and Foundation, Rochester, Minnesota, United States of America; 3 Basic Research Laboratory, Center for Cancer Research, National Cancer Institute, Bethesda, Maryland, United States of America; University of North Carolina School of Medicine, United States of America

## Abstract

We previously reported the identification of *TUSC1* (Tumor Suppressor Candidate 1), as a novel intronless gene isolated from a region of homozygous deletion at D9S126 on chromosome 9p in human lung cancer. In this study, we examine the differential expression of *TUSC1* in human lung cancer cell lines by western blot and in a primary human lung cancer tissue microarray by immunohistochemical analysis. We also tested the functional activities and mechanisms of *TUSC1* as a tumor suppressor gene through growth suppression *in vitro* and *in vivo*. The results showed no expression of *TUSC1* in *TUSC1* homozygously deleted cells and diminished expression in some tumor cell lines without *TUSC1* deletion. Interestingly, the results from a primary human lung cancer tissue microarray suggested that higher expression of *TUSC1* was correlated with increased survival times for lung cancer patients. Our data demonstrated that growth curves of tumor cell lines transfected with *TUSC1* grew slower *in vitro* than those transfected with the empty vector. More importantly, xenograph tumors in nude mice grew significantly slower *in vivo* in cells stably transfected with *TUSC1* than those transfected with empty vector. In addition, results from confocal microscopy and immunohistochemical analyses show distribution of *TUSC1* in the cytoplasm and nucleus in tumor cell lines and in normal and tumor cells in the lung cancer tissue microarray. Taken together, our results support *TUSC1* has tumor suppressor activity as a candidate tumor suppressor gene located on chromosome 9p.

## Introduction

Lung cancer is the most common form of cancer mortality in men and women in the world with an estimated 226,160 new cases and 160,340 deaths occurring in the United States in 2012 [1]. Lung cancer develops through a multistage process involving a variety of genetic and epigenetic changes in dominant oncogenes and tumor suppressor genes (TSGs) [2]. Non-small cell lung cancer (NSCLC) accounts for approximately 85% of all lung cancer subtypes with small cell lung cancer accounting for the remaining 15% [3], NSCLC is further subdivided into adenocarcinoma (39%), squamous cell carcinoma (21%), large cell carcinoma adenocarcinoma (3%), and uncommon types and combined types comprising the remaining 22% [3].

Alteration of chromosome 9p is implicated in a variety of tumor types including melanoma, non small cell lung carcinoma, breast cancer, leukemia, and hepatocellular carcinoma, clear cell renal cell carcinomas and gastrointestinal stromal tumors through chromosomal inversions, translocations, loss of heterozygosity (LOH) and homozygous deletion (HD). Genetic alterations of chromosome 9p occur early and frequently in lung cancer. These data suggest chromosome 9p contains a tumor suppressor locus (loci) critical in the development of several tumor types including lung [4]–[17]. Two candidate tumor suppressor loci were identified in the chromosome 9p21 region. One locus is *p16/CDKN2A*, which encodes the *p16* and *p14^ARF^* proteins. The other locus is *p15/CDKN2B* encoding the *p15* protein [7], [18], [19]. Since *p16/CDKN2A* is frequently inactivated genetically or epigenetically in cancer cells, the *p16/CDKN2A* locus is suspected to be a major tumor suppressor gene [20]–[25]. However, our previous studies in primary NSCLC, a large number of human lung cancer cell lines, and primary tumor samples identified a region of homozygous deletion (HD) at the microsatellite marker D9S126 which is distinct from the *p16/CDKN2A* locus and lies approximately 3.7 Mb proximal to *p16/CDKN2A*, [10], [14]. We had reported the identification of *TUSC1* (Tumor Suppressor Candidate 1) from this region and showed that expression of *TUSC1* was absent or diminished in cell lines with or without homozygous deletion of *TUSC1*. These findings prompted us to suggest that *TUSC1* may function as a tumor suppressor gene in lung tumorigenesis [14].

In this study, we have developed a C-terminal peptide antibody specific to *TUSC1* and stably transfected lung cancer cell lines (Nu6-1 and H290) in order to study *TUSC1’s* effect on cell growth of tumor cell lines with homozygous deletion of *TUSC1 in vitro* and tumor growth *in vivo* to characterize *TUSC1’s* potential tumor suppressing activity. We also examined the correlation between expression of *TUSC1* and survival times of lung cancer patients by immunohistochemical analysis of a human lung cancer tissue microarray. Our results demonstrated that cell growth curves of cells transfected with *TUSC1* grow slower *in vitro* than cells transfected with the empty vector. Moreover, subcutaneous injection of stably transfected cells containing *TUSC1* significantly suppresses growth of xenografts. The data also showed a trend towards increased survival times for lung cancer patients with higher levels of *TUSC1* expression. We were able to demonstrate localization of *TUSC1* protein in both the cytoplasm and nucleus in transfected cells, untransfected cells and primary tumor tissue. Taken together, we provide evidence that *TUSC1* functions as a tumor suppressor gene in tumor development and *TUSC1* may be a potential biomarker for diagnosis, prognosis and treatment. Future studies using *TUSC1* knock-out mice and identification of its interacting partner(s) and functional pathway(s) will address *TUSC1*’s physiological roles in tumor development.

## Materials and Methods

### Cell Culture, RNA Extraction and RT-PCR

The CHO cells were obtained from the American Type Culture Collection (ATCC). A squamous carcinoma cell line (SKMES-1) and three previously reported *TUSC1* homozygously deleted human lung cancer cell lines H290, Nu6-1 and NE18 were a gift of Dr. Steve Belinsky at Lovelace Respiratory Research Institute and were used for *in vitro* and *in vivo* experiments [26]–[28]. The genomic status of the *TUSC1, CDKN2A* and *CDKN2B* loci have been determined previously [14]. The cell lines were maintained at 37°C in RPMI 1640 medium (Gibco, Carlsbad, CA) supplemented with 10% fetal calf serum (FCS), 1% penicillin and streptomycin, and 5% L-glutamine (Gibco, Carlsbad, CA). Total RNA was isolated using TRIzol Reagent (Invitrogen, Carlsbad, CA) following the supplier’s protocol. RT-PCR for *TUSC1* was performed as described previously [14].

### Mutation Analysis

Mutational analysis was performed using primer sets covering the entire open reading frame of the *TUSC1* gene (GenBank: AY168647). Briefly, 50 ng of DNA from primary tumors and cell lines was used for amplification. The reaction conditions were optimized using the GC-RICH PCR System following the provided protocol (Roche, Indianapolis, IN). PCR products were purified by using a PCR purification kit (Qiagen, Valencia, CA) and then directly sequenced using a Bigdye terminator 1.1 and Applied Biosystems model 3130XL analyzer DNA (Applied Biosystems Inc, Foster City, CA). Sequencer DNA sequencing software was used to analyze and assemble sequences to determine nucleotide alterations.

### Antibody Production, Cell Lysates and Immunoblotting

Polyclonal specific antisera to TUSC1 were raised in rabbits by injecting a synthetic peptide corresponding to the carboxy-terminal sequence of the deduced TUSC1 protein (GenBank: AY168647) (Animal Pharmacy, Healdsburg, CA). The rabbit antibodies were affinity purified using the synthetic peptide coupled to Affigel-15 (Bio-Rad, Hercules, CA) [29]. Specificity of the TUSC1 antibody was determined by western blots with proteins from *TUSC1* homozygously deleted cells and proteins purified through ProBond™ Purification System (Invitrogen, Carlsbad, CA) from CHO cells stably transfected with *TUSC1* in pcDNA3.1/V5-His vectors (Invitrogen, Carlsbad, CA).

For Western blot analysis, protein lysates were prepared following the provided protocol (Thermo Fisher Scientific Inc, Rockford, IL). Proteins (25–30 µg) were loaded on a 10% SDS-polyacrylamide gel, followed by blotting on a nitrocellulose membrane (Invitrogen, Carlsbad, CA/Bio-Rad, Hercules, CA). Membranes were blocked for two hours with 5% nonfat milk in TBST buffer (0.1% Tween 20 in TBS) at room temperature, and the membrane was probed with the polyclonal antibody to TUSC1. After three washings with TBST, membranes were incubated with anti-rabbit secondary antibody (Cell Signaling, Danvers, MA) and washed five times with TBST at room temperature. The membranes were developed by SuperSignal@ West Pico Chemiluminescent Substrate Western blotting detection reagents for Kodak Biomax MR film exposure (Thermo Fisher Scientific Inc, Rockford, IL).

### Immunofluorescence and Immunohistochemistry Analyses

For immunofluorescence analysis, cells were seeded on coverslips in six-well plates one day before the experiment. Cells were fixed in 3% paraformaldehyde for 15 minutes at room temperature and washed two times (five minutes each) in PBS. Cells were then permeabilized with 0.1% Triton X-100 for five minutes, incubated with anti-*TUSC1* or anti-V5 antibody (Invitrogen, Carlsbad, CA) over night at 4°C followed by detection with FITC/or Texas Red-conjugated anti-rabbit IgG (Vector Laboratories, Burlingame, CA) for anti-TUSC1 antibody and Texas Red-conjugated anti-mouse IgG for anti-V5 antibody. Cells were analyzed with an Axiophot microscope equipped for immunofluorescence or an LSM S10 UV System for confocal image analysis (Zeiss, Thorword, NY). Immunohistochemical staining of normal human lung tissue and a lung cancer tissue microarray were conducted following the previously described protocol [30]. Detailed information about the clinical samples selection, tissue microarray, image analysis and immunohistochemistry scoring were reported previously [30]. Briefly, 10% horse serum was used for blocking. For antibody incubation, 1 ug/ml of *TUSC1* antibody was applied. After washing three times with PBS, the slide was incubated with biotinylated secondary antibodies (Vector Laboratories, Burlingame, CA) and then incubated for 45 minutes with avidin-biotin complex method reagent (Vectastain Elite ABC kit; Vector Laboratories, Burlingame, CA) and washed three times with PBS. The slide was then developed in liquid 3,3′-diaminobenzidine (DAKO, Carpinteria, CA) and lightly counterstained with Mayer’s hematoxylin, dehydrated, cleared, and mounted with resinous mounting medium.

### Construction of TUSC1 Expression Vectors and Generation of Stably Transfected Cell Lines

The open reading frame of the *TUSC1* was amplified using *TUSC1* specific primers (data not shown). Fragments were cloned into pcDNA3.1 or pcDNA3.1/V5-His vectors (Invitrogen, Carlsbad, CA) for transfection experiments. The inserted sequences and orientations were sequence verified.

For transfection, CHO, 9HTE, Nu6-1, and H290 cell lines were grown to 80% to 90% confluence for cell transfection. The transfections were performed with Lipofectamine™ 2000 following the provided protocol (Invitrogen, Carlsbad, CA). To establish stably transfected cell lines, cells were selected using geneticin treatment (G418, 500–800 µg/ml, Invitrogen, Carlsbad, CA) 48 hours after transfection for two to three weeks. A pool of selected cells was re-cultured under continuous selection with geneticin. The protein expression levels were determined by Western blot analyses to verify the expression of *TUSC1* in the transfectants.

### Cell Proliferation *In Vitro* and Tumor Growth *In Vivo*

For *in vitro* analysis, the cells were trypsinized and cell numbers were measured in a Coulter Counter (ZM; Scientific Instruments, Hialeah, FL). Cell proliferation was measured at 24-hour intervals for five days. The results represent the mean of three independent experiments.

All animal research was conducted in facilities accredited by the Association for Assessment and Accreditation of Laboratory Animal Care Institutional (AAALAC) at NIH. Animal studies were performed in accordance with U.S. National Institutes of Health guidelines and were conducted under a protocol approved by NIH Animal Care Use Committee. For xenograft experiments, ten mice for each cell line were used to study tumor development *in vivo*. In each animal, 1×10^6^ stably *TUSC1* transfected tumor cell lines, either Nu6-1 or H290, were injected on the left side with the corresponding tumor cell line transfected with empty vector injected on the right side of nude mice as controls. Prior to injection, the mice were anesthetized using isoflurane. Tumor size was measured weekly and approximate tumor volumes were determined by multiplying tumor height×length×width. If the tumor measurements were at or larger than 2 cm, the mice were euthanized by carbon dioxide inhalation in a chamber attached to house CO_2_. Humane endpoints were observed if other health issues caused the mice to experience symptoms such as rapid weight loss, debilitating diarrhea, labored breathing, bleeding from any orifice, self-induced trauma, impaired mobility, etc.

### Statistical Analysis

Results of the experiments done *in vitro* and *in vivo* are reported as mean ± s.d. Statistical comparisons between test and control samples were evaluated by Student's *t*-test and the significance was set as *p*<0.05. The log-rank test was used for comparing survival distributions between positive and negative groups of staining on tumor tissue microarrays and Kaplan-Meier curves were plotted for the two groups.

## Results

### Analysis of *TUSC1* Somatic Mutation

Our previous results located the *TUSC1* gene in a homozygous deletion region on chromosome 9p and demonstrated reduced expression in lung cancer cell lines [14]. These results indicated *TUSC1* may be a candidate TSG and genetically altered. To test this hypothesis, we performed mutation analysis of the open reading frame of *TUSC1* **(**GenBank: AY168647**)** on 97 genomic DNA samples including 45 cancer cell lines (22 lung, 17 melanoma and six colon), 14 matched NSCLC cell line pairs, six matched Small Cell Lung Cancer (SCLC) cell line pairs and six matched SCLC primary tumors. Five previously identified single-nucleotide polymorphisms (SNPs) within the open reading frame (ORF) of *TUSC1* were identified in these samples. Results from a database search identified numerous SNPs located in the 3′ and 5′ untranslated regions (UTRs) as well as seven SNPs (three nonsynonymous coding and four synonymous coding variants) located in the ORF of *TUSC1* (The Wellcome Trust Sanger Institute, http://www.sanger.ac.uk/resources/databases). Three nonsynonymous SNPS (rs72631813, rs34498078 and rs72631815) and two synonymous SNPs (rs72631814 and rs35110225) were found in our samples. We observed the SNPs in multiple samples and some samples had multiple SNPs (data not shown). Furthermore, the SNPs at nucleotides 189, 358, 378 and 613 occurred at a high frequently in the samples tested for this report. However, we and others did not detect nonsense mutations in *TUSC1* as mutational analysis reported for biliary tract, breast, central nervous system, large intestine and pancreas also did not identify any mutations [31]–[34]. These results indicate somatic mutation of the *TUSC1* was not a major event.

### Generation of Cell Lines Expressing TUSC1 and a Polyclonal Antibody for TUSC1

**Figure 1:**
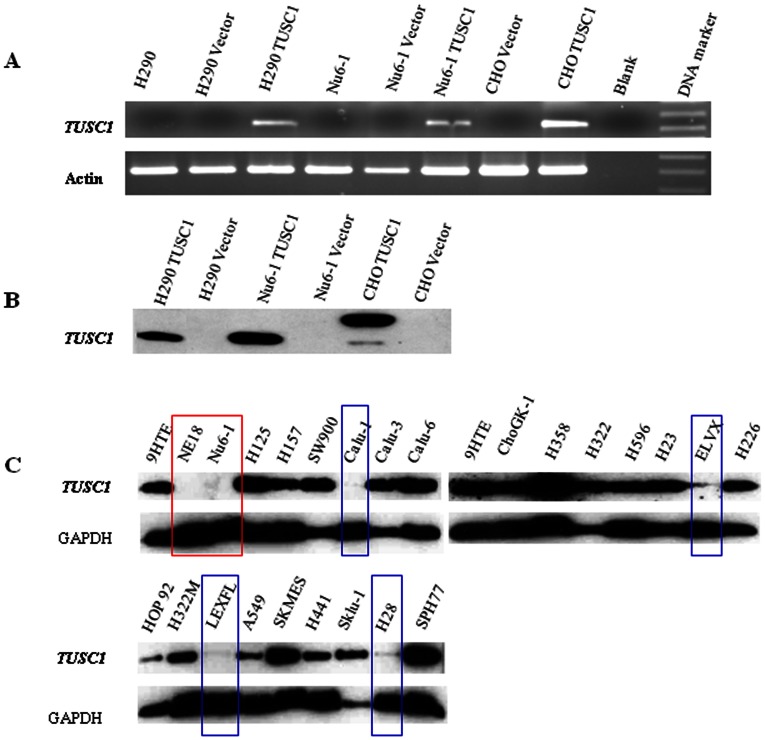
Expression of *TUSC1* mRNA and protein in cell lines. (A–B) The tumor cell lines (H290 and Nu6-1) were stably transfected with the *TUSC1* gene in pcDNA3.1 vector and CHO cells were transfected with *TUSC1* in pcDNA3.1/V5-His vector. *TUSC1* mRNA was amplified (RT-PCR) and exogenous protein (Western blot) was detected in all stably transfected cell lines containing *TUSC1* expression clones but not the parental cell lines transfected with empty vector. (C) Western blot analysis of endogenous TUSC1 proteins in tumor cell lines. Red box: Cell lines with homozygous deletion of *TUSC1*; Green box: cell lines with reduced TUSC1 expression but without *TUSC1* deletion. As an internal control for the amount of protein loaded, the same membrane was incubated with Anti-GAPDH antibody.

To test the functional activity of *TUSC1*, we generated stably transfected cells by transfecting expression vectors containing the *TUSC1* open reading frame into cells harboring a homozygous deletion of the *TUSC1* gene (Nu6-1 and H290). The expression of *TUSC1* mRNA and protein in the pooled transfected cell cultures was verified by RT-PCR and Western blot (Figure 1A, B). As expected, *TUSC1* expression was only detected in the cells transfected with *TUSC1* expression vectors, and not in the parental cell lines transfected with either empty vector or not transfected. The results demonstrated stable expression of exogenous *TSUC1* and provided an important reagent to test the specificity of the *TUSC1* antibody.

A polyclonal antibody was generated for *TUSC1* using four peptides based on the predicted TUSC1 amino acid sequence (Gene Bank access no: AY168647). The peptides were synthesized and used to immunize rabbits. Polyclonal antibodies were affinity purified and the specificity and sensitivity were verified by a series of western blots, using protein extracts from CHO cells stably transfected with *TUSC1* in pcDNA3.1/V5-His vectors and proteins from cells with or without the *TUSC1* homozygous deletion (data not shown). We demonstrated that a C-terminal peptide antibody was specific as it detected a single band with the expected molecular weight of *TUSC1* in the protein lysates isolated from CHO cells stably transfected with the *TUSC1* expressing vector. The corresponding protein band was detected in cell lysates from *TUSC1* homozygously deleted cells (Nu6-1, H290) containing the *TUSC1* expression vector. However, no signals were detected in the protein lysates from the parental Nu6-1 and H290 cell lines transfected with empty vector (Figure 1B).

### Reduced Expression of TUSC1 Correlates with Poor Survival Rates in Patients with NSCLC

**Figure 2:**
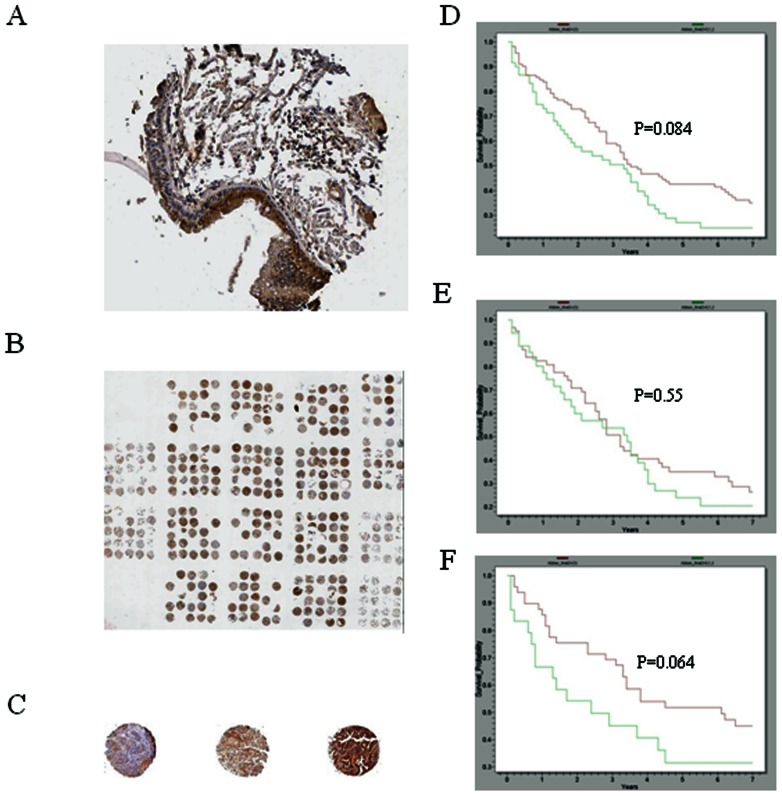
Representative immunohistochemical analysis using the TUSC1 antibody in normal and primary lung tissues. (A) Bronchioepithelium of Normal Lung. The basal layer was not positive for TUSC1 but differentiated cells in the upper layers of epithelium have high TUSC1 expression. Distribution of TUSC1 staining was cytoplasmic, nuclear and nuclear/cytoplasmic in the cells. (B, C) Lung cancer tissue microarray stained with *TUSC1* antibody and representative tissue cores (1–3) for each level of staining intensity. (D–F) Kaplan-Meier survival plots for the overall patient (D), adenocarcinoma (E) and squamous cell carcinoma (F). Survival status and the associated *P* value are indicated. Red line represents tumors with higher expression levels of *TUSC1* (score: 3), green line represents tumor with lower expression levels of *TUSC1* (score: 0–2).

**Figure 3:**
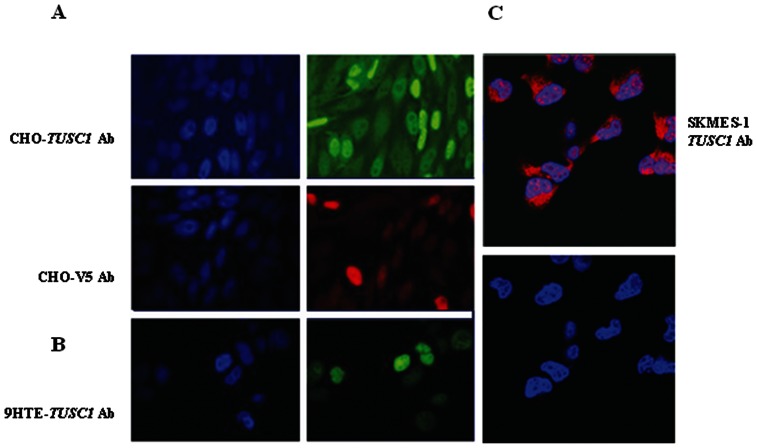
Immunofluorescent staining by confocal microscopy. Localization of *TUSC1* in stable transfectants of CHO and 9HTE cell lines. (A) Stably transfected CHO cells were incubated with V5 or *TUSC1* antibodies. (B–C) Un-transfected 9HTE and SKEMS-1 lung cancer cells were incubated with *TUSC1* antibody. Subcellular distributions of *TUSC1* proteins are cytoplasmic and nuclear. V5 antibody was detected with Texas Red-conjugated anti-mouse IgG (Red) and *TUSC1* antibody was detected with either Texas Red-conjugated anti-rabbit IgG (Red) or by FITC-conjugated anti-rabbit IgG (Green).

We previously reported the expression of *TUSC1* mRNA in normal adult tissues tested by northern blot, and a lack of expression in cells bearing homozygous deletion of *TUSC1,* as well as reduced expression of *TUSC1* in other cell lines without *TUSC1* deletion [14]. In western blot, expression of TUSC1 was not detected in cell lines (Nu6-1 and NE18) bearing homozygous deletion of the TUSC1 gene and had diminished expression of TUSC1 in cell lines without the deletion of TUSC1 gene (Figure 1C). These findings led us to test expression levels in primary tumors and to correlate these levels with patient outcome in order to further test *TUSC1’s* potential tumor suppressor activity. We evaluated the expression patterns of TUSC1 in a previously described primary human lung cancer tissue microarray by immunohistochemistry (IHC) [30]. The tissue microarray contains 150 adenocarcinomas and 150 squamous cell carcinomas. In normal bronchial epithelial cells of the lung, TUSC1 expression was localized in the differentiated cells of the upper cell layers but expression was reduced or absent in the basal cell layers. We also observed three TUSC1 staining patterns including cytoplasmic, nuclear and nuclear/cytoplasmic patterns (Figure 2A). However, the proportion of positive staining of TUSC1 in both cytoplasm and nucleus was not equal in all the samples, and there were various levels of staining intensity (Figure 2A–C). We scored the tissue microarray samples based on the expression level and staining intensity of TUSC1 using a method described previously [30]. Following scoring of the tumors, the results suggested a correlation between increased expression of TUSC1 and longer survival times in patients having the higher score [30]. The correlation was somewhat stronger in patients with squamous carcinomas (Figure 2F) and the results showed a trend towards statistical significance (p = 0.064). We also determined the cellular location of TUSC1 by immunofluorescence with confocal microscopy on both untransfected and stably transfected cell lines. The results showed exogenous and endogenous TUSC1 proteins are located in both the cytoplasm and nucleus of CHO transfected cells as well as in untransfected 9HTE cells and a lung cancer cell line (SKMES-1) endogenously expressing TUSC1 (Figure 3A–C). The results suggest TUSC1 may function in multiple pathways depending on subcellular localization.

### Restoring Expression of the TUSC1 Gene Reduces Tumor Cell Growth *in vitro* and Suppresses Tumor Formation *in vivo*

**Figure 4:**
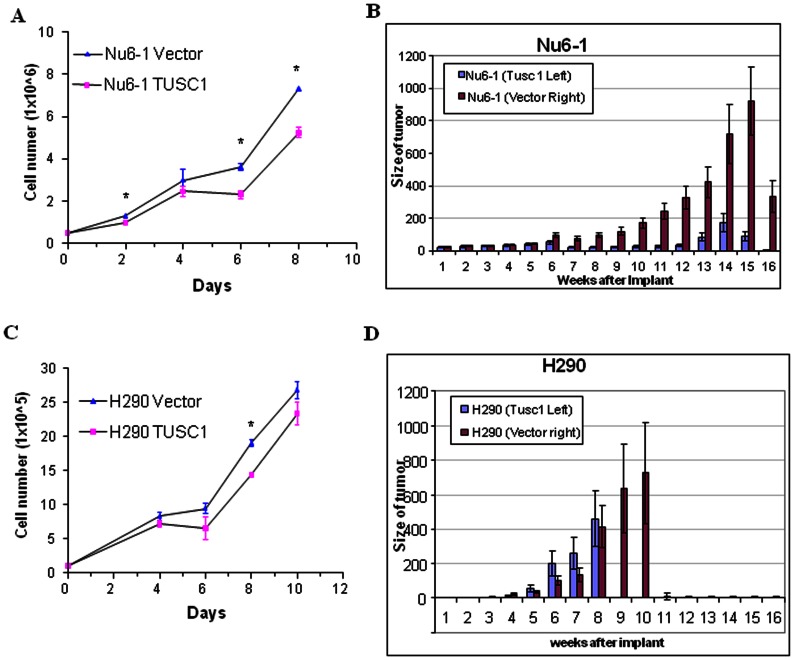
Overexpression of *TUSC1* reduces cell growth and tumorigenicity of lung cancer cells. (A, B) *In vitro* cell growth of cancer cell lines (Nu6-1 and H290) transfected with *TUSC1*. (C, D) Tumor volume of subcutaneously injected stably transfected cancer cells with *TUSC1* in nude mice *in vivo*. Restoration of *TUSC1* expression in cell lines with *TUSC1* homozygous deletion suppressed cell growth and tumor development compared to the parental cell lines transfected with the empty vector (p<0.05 ).

Restoring expression of *TUSC1* in homozygously deleted cells was achieved by generating stably transfected cells. The expression levels of *TUSC1* were verified by RT-PCR analysis and western blot (Figure 1B). Results from cell proliferation experiments showed the cell growth rate in the stably transfected cells, Nu6-1 and H290, with *TUSC1* expressing vector grew slower than cells transfected with empty vector (Figure 4A, C). These results suggest that exogenous expression of the *TUSC1* gene can reduce tumor cell line growth *in vitro*.

We further determined whether the inhibitory effects of the *TUSC1* gene on tumor cell proliferation *in vitro* could be demonstrated through tumor growth *in vivo* in nude mice. We generated tumor xenografts by subcutaneously injecting 1×10^6^ *TUSC1* stably transfected Nu6-1 and H290 cells or empty vector containing cells into nude mice to evaluate the efficacy of the gene in suppressing tumor growth. *TUSC1* transfected cells were injected on the left side of each animal and the corresponding vector control cells were injected on the right side of the same animal. The xenografts were monitored for three months for tumor size and the growth of tumors. Average tumor volumes from the cells transfected with *TUSC1* were compared with the average tumor volume from the parental cell line transfected with the empty expression vector. The results show that *TUSC1* significantly suppresses tumor growth (p<0.05, Figure 4B). These data are not only consistent with the data from the *in vitro* study, but also demonstrate that *TUSC1* is effective in reducing tumor cell line growth *in vitro* and *in vivo*.

## Discussion

It is well known that genetic alterations of chromosome 9p occur in multiple types of human cancers including lung. Identification of functional tumor suppressor(s) on chromosome 9p will provide opportunities to develop biomarkers and innovative therapeutic strategies urgently needed for cancer diagnosis, prognosis and treatment. We had previously reported a putative tumor suppressor gene, *TUSC1*, which resides in a region of homozygous deletion at marker D9S126 and we had demonstrated reduced expression of *TUSC1* mRNA in human lung cancer cell lines [14]. In the current study, we were able to show reduced and differential expression of *TUSC1* protein in lung cancer cell lines and primary lung cancer tissue samples using a TUSC1 specific polyclonal antibody (Figures 1, 2). Most importantly, introducing the *TUSC1* gene into tumor cell lines harboring a homozygous deletion of *TUSC1* (Nu6-1 and H290) had the effect of inhibiting cell proliferation *in vitro* and reducing tumor growth *in vivo* (Figure 4). These results further support our previous hypothesis that *TUSC1* may play an important role in lung tumorigenesis and function as a tumor suppressor gene (14). Moreover, our data indicate higher levels of TUSC1 expression is correlated with increased survival times for lung cancer patients (Figure 2). This lead us to suggest that expression levels of *TUSC1* may be a potential biomarker for prognosis, although, more studies will be required with larger sample sizes and detailed clinical data to support this concept. In addition, we also demonstrated TUSC1 resides in both the cytoplasm and nucleus of normal cells, tumor cell lines and tumor tissue microarrays (Figures 2, 3) suggesting TUSC1 may participate in multiple functional pathways for its physiological function.

The mechanism by which *TUSC1* expression is reduced in lung cancer cells and tumor tissues, and the mechanism by which *TUSC1* governs the inhibition of NSCLC cell growth remain to be fully elucidated. To date we have not detected hypermethylation of the promoter region of the *TUSC1* gene in the cell lines with the diminished expression of *TUSC1* or increased apoptosis by FACS analysis through over-expression of *TUSC1* (data not shown). Moreover, we and others did not detect somatic mutations in the *TUSC1* ORF in the samples tested or as reported in the literature [32]–[34]. However we were able confirm five SNPs also reported in SNP database (The Wellcome Trust Sanger Institute, http://www.sanger.ac.uk/resources/databases). Additionally, the relationship between the SNPs and effects on protein structure or function of the gene remain to be determined. Interestingly, one SNP, rs13290968, in the *TUSC1* gene was recently reported to be associated with a pigmentation phenotype and tanning ability among cutaneous malignant melanoma patients [17]. Future directions include testing whether nonsynonymous SNPs can alter the protein structure and/or the binding ability of *TUSC1* to its potential protein partner(s) as well as cell growth characteristics by overexpressing the mutated ORF in *TUSC1* homozygously deleted cells. These results may help to explain diminished *TUSC1* expression and why mutation of *TUSC1* was not seen as a frequent event in tumorigenesis. Additionally, we noted a significant reduction in tumor frequency and volume in the animals subcutaneously injected with *TUSC1* stably transfected Nu6-1 cells as opposed to H290 cells and restoration of *TUSC1* expression appears to have more effects *in vivo* than *in vitro* (Figure 4). These results suggest that the reduction of tumor and cell growth by *TUSC1* might not be solely due to the exogenous *TUSC1* expression. One possible explanation may be that the *in vivo* microenvironment activates potential *TUSC1* pathway(s) more effectively than in the *in vitro* setting. Toward this goal, we are identifying and validating potential binding partners by yeast two-hybrid analysis.

Taken together, we have provided further evidence that *TUSC1* expression is downregulated in lung cancer cell lines and a trend towards higher expression of *TUSC1* is correlated with longer survival times for lung cancer patients. Restoration of *TUSC1* expression in lung cancer cells is followed by the suppression of tumorigenicity *in vivo* and slows cell growth *in vitro*, suggesting *TUSC1* functions as a tumor suppressor gene. Further study into *TUSC1’s* activity by interacting with key components of survival pathways and other interacting proteins, and generating a knock-out mouse model for *TUSC1,* will provide more evidence to support *TUSC1’s* function as a tumor suppressor gene and help us to address its physiological functions. Future studies to elucidate the effect of nonsynonymous SNPs will be interesting to understand if there is a cancer-associated SNP(s) and their effects on *TUSC1* activity. The results may also provide a strategy for the prevention, early detection, diagnosis, and treatment for lung cancer and other human cancers related to the loss of chromosome 9p21 region.
